# Onlay Versus Inlay Biceps Tenodesis for Long Head of Biceps Tendinopathy: A Systematic Review and Meta-analysis

**DOI:** 10.5435/JAAOSGlobal-D-22-00255

**Published:** 2022-12-09

**Authors:** Garrett R. Jackson, Joshua Meade, Kyle Coombes, Bradley L. Young, Nady Hamid, Dana P. Piasecki, James E. Fleischli, David P. Trofa, Bryan M. Saltzman

**Affiliations:** From the Department of Orthopaedic Surgery, Rush University Medical Center, Chicago, IL (Dr. Jackson); the OrthoCarolina, Charlotte, NC (Mr. Meade, Dr. Young, Dr. Hamid, Dr. Piasecki, Dr. Fleischli, and Dr. Saltzman); the Department of Orthopaedic Surgery, Atrium Health Musculoskeletal Institute, Charlotte, NC (Mr. Meade, Dr. Young, Dr. Hamid, Dr. Piasecki, Dr. Fleischli, and Dr. Saltzman); the American University of the Caribbean School of Medicine, Cupecoy, Sint Maarten (Mr. Coombes); and the Department of Orthopedics, Columbia University Medical Center, New York, NY (Dr. Trofa).

## Abstract

Proximal biceps tenodesis is a common surgical treatment of tendinosis of the long head of the biceps tendon. Two of the most common techniques incorporate onlay and inlay fixation methods, which can be done arthroscopically or open and in a variety of anatomic locations. The purpose of this meta-analysis was to compare the clinical outcomes between onlay versus inlay humeral fixation for biceps tenodesis for long head of the biceps tendon pathology.

**Methods:**

A literature search was conducted using PubMed, EMBASE, and Cochrane Library. Only studies reporting outcomes and complications after onlay and inlay biceps tenodeses were included.

**Results:**

Six studies with a total of 418 patients (252 onlay, 166 inlay) with a mean age of 56.84 years were included. Visual analog pain scale scores, Constant score, and American Shoulder and Elbow Surgeons shoulder score did not differ. “Popeye” deformity was found in 17 patients (7.80%) in the onlay group and in 15 patients (11.28%) in the inlay group (odds ratio, 0.28; *P* = 0.07). No difference in postoperative cramping or failure rates was found.

**Conclusion:**

Both onlay and inlay biceps tenodeses result in improved clinical outcomes and are at low risk of Popeye deformities, with no statistically significant differences between either method. Additional studies are required to assess the clinical significance of these differences.

Tendinosis of the long head of the biceps tendon (LHBT) arises from various causes related to instability or trauma and is a common cause of anterior shoulder pain.^1^ Biceps tenodesis and tenotomy have been widely accepted as the treatments of biceps tendinopathy that is recalcitrant to nonsurgical management.^1,2,3,4,5^ When compared with biceps tenotomy, tenodesis of the LHB has gained popularity because of its ability to preserve function of the LHBT; maintain its length-tension relationship; and reduce cramping, pain, loss of strength, and cosmetic defects.^1,4,6,7,8,9,10,11,12^

Several tenodesis techniques have been developed to treat patients with LHBT pathologies. These techniques can be divided into onlay, inlay, and soft-tissue fixation.^2,13,14,15,16^ Soft-tissue fixation has markedly decreased clinical and structural outcomes compared with the onlay and inlay bony fixation techniques.^15^ The inlay technique is an intraosseous tenodesis that secures the LHBT within a subcortical bone socket by using interference screws or bicortical suspensory devices while the onlay technique secures the tendon to the cortical surface by suture anchors or unicortical suspensory devices. Many studies have reported the inlay technique to have increased biomechanical strength when compared with the onlay technique; however, it is associated with increased risk of torsional fracture of the proximal humerus secondary to the stress created from the drill hole used to create the bone socket.^2,17,18,19,20,21,22^ The onlay technique results in decreased revision rates compared with the inlay technique.^23^ Current literature does not offer consensus regarding whether onlay or inlay tenodesis is the preferred surgical technique for a proximal biceps tenodesis.

The purpose of this meta-analysis was to compare the clinical outcomes between onlay versus inlay humeral fixation for biceps tenodesis for LHBT pathology. The authors hypothesized that both onlay and inlay biceps tenodeses for long head of biceps tendinopathy demonstrate no notable difference in clinical outcomes and “Popeye” deformities.

## Methods

### Search Strategy and Data Extraction

The literature search and data extraction were conducted using the guidelines of the 2020 Preferred Reporting Items for Systematic Reviews and Meta-Analysis. In January 2021, a comprehensive database search was conducted using PubMed, EMBASE, and Cochrane Library. The reference lists of the original studies were searched for additional studies. The search criteria included the keywords “biceps” and “tenodesis.”

**Figure 1:**
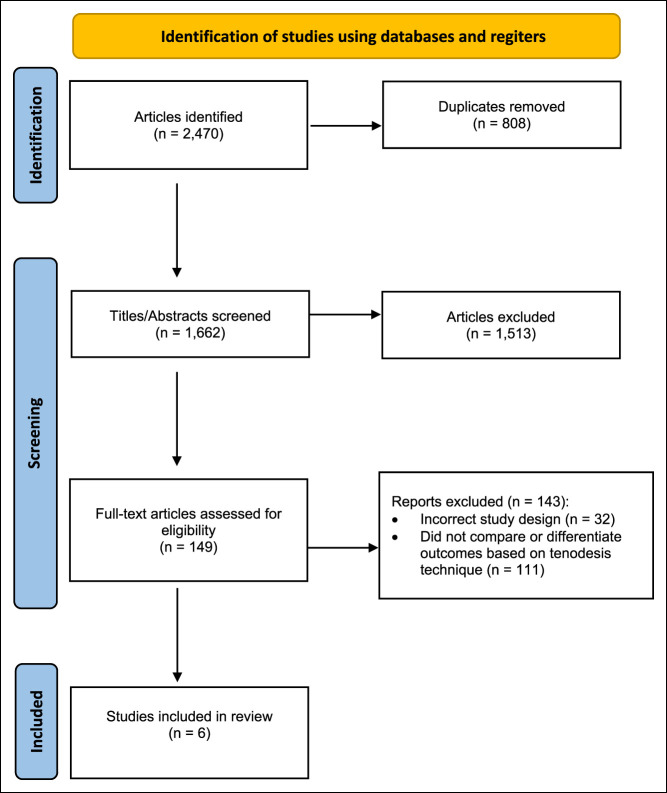
Chart showing Preferred Reporting Items for Systematic Reviews and Meta-Analysis (PRISMA) flow diagram.

The initial search yielded 2470 articles. Articles in languages other than English, biomechanical studies, letters to editors, non–full-text articles, case reports, meta-analyses, and review articles were excluded. Of the 2470 articles, 808 were duplicates. The title and abstract screening included 1662 articles, of which 1513 were excluded. Only 149 full-text articles were evaluated for eligibility (Figure 1). After the full-text screening, six studies met our criteria comparing inlay with onlay biceps tenodesis. The data were extracted from the results of the included studies and was entered into a Microsoft Excel version 16.63 (Redmond) spreadsheet for additional analysis. The selected articles were not blinded for the author, affiliation, or source.

Two independent authors (G.R.J. and J.M.) reviewed the abstracts, followed by a full-text review for the identified articles. To avoid duplicate patient cohorts, multiple studies that included the same authorship were flagged and only the most recent article was included for our data extraction.

### Outcomes Measures

The following outcomes measures were analyzed: American Shoulder and Elbow Surgeons shoulder score (ASES), visual analog pain scale (VAS), Constant score (CS), the presence of a Popeye deformity, and postoperative cramping. Only five of the included studies reported on the presence of a Popeye deformity while only two studies reported on postoperative cramping.

### Methodological Quality Assessment

Methodological quality assessment of the selected articles was conducted using the Coleman score, which evaluates the quality of randomized and nonrandomized orthopaedic studies. This score includes a list of 10 criteria, with a final score ranging from 0 to 100. Higher scores indicate the absence of biases, whereas lower scores equate to increased biases. The scores are categorized into excellent (85 to 100), good (70 to 84), fair (50 to 69), and poor (<50). Each selected study was given a calculated score by reviewers.

### Data Analysis

The meta-analysis software Review Manager 5.4.1 (Cochrane Collaboration) was used for the data analysis. Each outcome measure was depicted in a forest plot. The forest plots indicated the standardized mean differences, individual study weights, and 95% confidence intervals for each article. The event outcomes for postoperative Popeye deformities and failure rates were individually depicted in forest plots indicating the odds ratios (ORs), individual study weights, and 95% confidence intervals. Failure was defined by visualizing the failure of the tendon fibers to trace longitudinally within the intertubercular groove at the insertion site as seen on MRI or ultrasonography. To measure the heterogeneity of the included studies, a chi square index was used. Owing to the presence of a high level of heterogeneity, a random-effects meta-analysis was used because of its ability to weigh studies more equally. To analyze publication bias of the included studies, a funnel plot was used. A *P* value of < 0.05 indicated statistical significance.

## Results

Six studies with a total of 418 patients met our search criteria. Of the 418 patients, 252 received an onlay biceps tenodesis using suture anchors and 166 received an inlay biceps tenodesis using an interference screw, with a mean patient age of 56.84 years (219 males, 187 females). Eighty-nine patients received an open tenodesis (89 inlay, zero onlay); 329 patients received an arthroscopic tenodesis (77 inlay, 252 onlay). A suprapectoral technique was used in 180 patients (70 inlay, 143 onlay) while a subpectoral technique was used in 143 patients (89 inlay, 54 onlay). Patients included in this analysis were treated with concomitant procedures such as rotator cuff repairs and débridement, labral repairs and débridement, subacromial decompressions, and distal clavicle excisions. The minimum length of follow-up was 6 months for one study, 12 months for five studies, and 24 months for the study by Park et al.^14^ Table 1 summarizes the characteristics of each study. Of the six included articles, three are of Level II evidence (one prospective, two randomized control trials) and three are of Level III evidence and retrospective.^2,13,14,16,24,25^ The Coleman score ranged between 53 and 74, indicating a fair quality of evidence.

**Table 1:** Study Characteristics

Author	LoE, Study	CS	No. of Patients	Mean age (range), yr	Sex(M/F), (n)	Mean Follow-up (range), mo	Tenodesis Fixation	Open/Arthroscopic (n)	Suprapectoral/Subpectoral, n	Outcomes	Complications (n)
Haidamous et al.^2^	III, retrospective cohort	55	Onlay: 53 Inlay: 37 Total: 90	Onlay: 63.1 (44-85) Inlay: 60.2 (43-76)	Onlay: 34/19 Inlay: 21/16	Onlay: 14.1 (12-25) Inlay: 18.2 (12-27)	Onlay: Suture anchor Inlay: Interference screw	Onlay: 0/53 Inlay: 0/37	Onlay: 53/0 Inlay: 37/0	Forward flexion, external rotation, internal rotation, VAS, ASES, SSV, return to activity, and satisfaction	Onlay: Popeye deformity (5), cramping (2), and revision surgery (0) Inlay: Popeye deformity (10), cramping (4), and revision surgery (4)
Millet et al.^25^	III, retrospective cohort	53	Onlay: 54 Inlay: 34 Total: 88	Onlay: 51 (22-77) Inlay: 51 (22-77)	Onlay: N/A Inlay: N/A Total: 57/31	Onlay: 13 Inlay: 13	Onlay: Suture anchor Inlay: Interference screw	Onlay: 0/54 Inlay: 34/0	Onlay: 0/54 Inlay: 0/34	VAS, ASES, and CS	Onlay: Postoperative groove Inlay: Postoperative groove tenderness (1)
Yi et al.^16^	III, retrospective cohort	59	Onlay: 36 Inlay: 35 Total: 71	Onlay: 54.32±1–0.73 Inlay: 53.46 ± 8.45	Onlay: 16/20 Inlay: 17/18	Onlay: 21.45 ± 1.24 Inlay: 21.03 ± 2.42	Onlay: Suture anchor Inlay: Interference screw	Onlay: 0/36 Inlay: 35/0	Onlay: 36/0 Inlay: 0/35	UCLA, CS, ASES, VAS, forward flexion, external rotation, abduction, and internal rotation	Onlay: Synovitis (3) Inlay: None reported
Franceschetti et al.^13^	II, randomized control trial	67	Onlay: 20 Inlay: 20 Total: 40	Onlay: 57.9 ± 3.2 (56-63) Inlay: 58.5 ± 3.5 (55-64)	Onlay: 13/7 Inlay: 12/8	Onlay: 12 Inlay: 12	Onlay: Suture anchor Inlay: Interference screw	Onlay: 0/20 Inlay: 20/0	Onlay: 20/0 Inlay: 0/20	VAS, CS, SST, and LHB score	Onlay: Popeye deformity (1) Inlay: None reported
Samargandi et al.^24^	II, prospective comparative	70	Onlay: 55 Inlay: 7 Total: 62	Onlay: 55.6 Inlay: 52.2	Onlay: 26/29 Inlay: 7/0	Onlay: 7.3 (median) Inlay: 7.3 (median)	Onlay: Suture anchor Inlay: Interference screw	Onlay: 0/55 Inlay: 0/7	Onlay: Not given Inlay: Not given	VAS, SSV, and CS	Onlay: Popeye deformity (11) and cramping (2) Inlay: Popeye deformity (5)
Park et al.^14^	II, randomized control trial	74	Onlay: 34 Inlay: 33 Total: 67	Onlay: 62.4 ± 8.2 Inlay: 61.2 ± 7.2	Onlay: 15/19 Inlay: 13/20	Onlay: 26.6 ± 5.3 Inlay: 28.8 ± 7.3	Onlay: Suture anchor Inlay: Interference screw	Onlay: 0/34 Inlay: 0/33	Onlay: 34/0 Inlay: 33/0	VAS, ASES, SST, CS, KSS, LHB score, and forward flexion. External rotation and internal rotation	Onlay: Failure (2) Inlay: Failure (7)

ASES = American Shoulder and Elbow Surgeons shoulder score, CS = Constant score, SSV = Subjective Shoulder Score, SST = Simple Shoulder Test, KSS = Knee Society Score, LHB = Long Head of the Biceps, UCLA = University of California at Los Angeles, VAS = visual analog pain scale

### American Shoulder and Elbow Surgeons Shoulder Score

**Figure 2:**
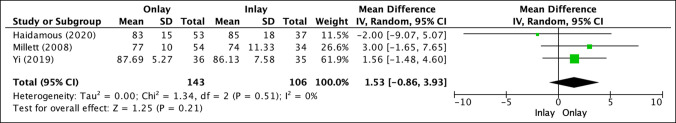
Illustration depicting the forest plot for American Shoulder and Elbow Surgeons shoulder score (ASES).

The American Shoulder and Elbow Surgeons shoulder score was reported in three studies (Figure 2).^1,2,16,25^ Onlay biceps tenodesis (143 patients) showed improved discrete postoperative results (84.31 ± 7.65) versus inlay biceps tenodesis (106 patients; 82.77 ± 7.65), which was not statistically significant (mean difference, 1.53; *P* = 0.21).

### Visual Analog Pain Scale

**Figure 3:**
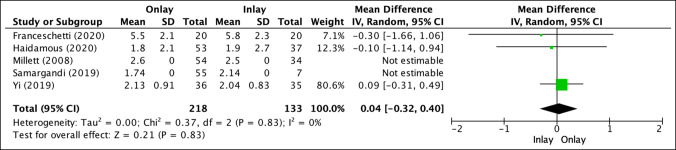
Illustration depicting the forest plot for visual analog pain scale (VAS).

In five studies that reported VAS (Figure 3), onlay biceps tenodesis (218 patients) showed increased discrete postoperative results (2.33 ± 1.14) versus inlay biceps tenodesis (133 patients; 2.29 ± 0.56), which was not statistically significant (mean difference, 0.04; *P* = 0.83).^2,13,16,24,25^

### Constant Score

**Figure 4:**
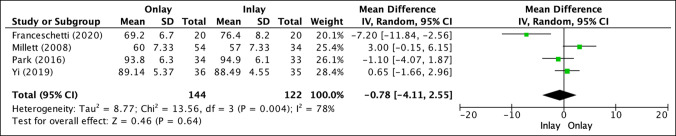
Illustration depicting the forest plot for Constant score (CS).

CS was reported in four studies (Figure 4).^13,14,16,25^ Inlay biceps tenodesis (122 patients) showed increased postoperative results (79.74 ± 6.39) versus onlay biceps tenodesis (144 patients; 78.95 ± 6.38), which was not statistically significant (mean difference, −0.78; *P* = 0.64).

### Popeye Deformity

**Figure 5:**
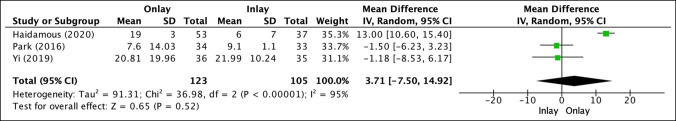
Illustration depicting the forest plot for Popeye deformity.

In the five studies that reported Popeye deformity (Figure 5), inlay biceps tenodesis showed increased postoperative Popeye deformities (15 of 133 patients; 11.28%) versus onlay biceps tenodesis (17 of 218 patients; 7.80%), which was found not to be statistically significant (OR, 0.28; *P* = 0.07).^2,13,16,24,25^

### Postoperative Cramping

**Figure 6:**
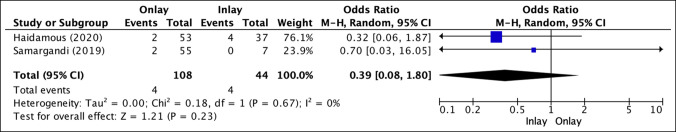
Illustration depicting the forest plot for postoperative cramping.

In the two studies that reported postoperative cramping (Figure 6), inlay biceps tenodesis showed an increased incidence of cramping (4 of 44 patients; 9.1%) versus onlay biceps tenodesis (4 of 108 patients; 3.7%), which was not statistically significant (OR, 0.39; *P* = 0.23).^2,24^

### Failed Repairs

**Figure 7:**
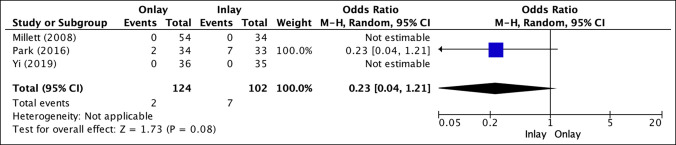
Illustration depicting the forest plot for failure of fixation.

Three studies reported failure of fixation (Figure 7).^14,16,25^ The inlay tenodesis reported an increased failure of fixation at 6.86% (7 of 102 patients) versus the 1.61% (2 of 124 patients) seen with the onlay tenodesis, which was not statistically significant (OR, 0.23; *P* = 0.08). Failure of fixation was determined by MRI or ultrasonography visualizing failure of the tendon fibers to trace longitudinally within the intertubercular groove at the insertion site.^14^

## Discussion

This meta-analysis aimed to compare outcomes and complications between onlay and inlay biceps tenodeses, but found no statistically significant difference in ASES, VAS, CS, Popeye deformities, or failure rates between the two techniques. These findings were consistent with the initial hypothesis proposed by the authors.

Regardless of whether the biceps tenodesis was done using an onlay or inlay technique, both methods report improved outcomes, with few complications.^2,13,14,25,26^ Although this analysis showed no statistical difference in Popeye deformities between the two techniques, a recent study of 37 patients reported an increased number of deformities with the inlay technique (27%) when compared with the onlay technique (9.4%).^2^ These findings were greater than the findings within this analysis in which 11.28% of inlay patients and 7.80% of onlay patients reported Popeye deformities, which were not statistically significant. The increased incidence of Popeye deformities seen in the inlay group is thought to be secondary to interference screws cutting into the tendon during insertion into the bone socket.^2,14,27^

An additional reported disadvantage of using the inlay technique is the need to create the bone socket. The increased size of the socket has been associated with a greater risk of torsional fracture of the proximal humerus.^9,22,27^ Creating this socket has also been found to increase the risk of causing musculocutaneous neuropathy and increased risk of infections with subpectoral fixation; ^28‐30^ however, these complications were not reported in the included studies of this meta-analysis.

Historically, biomechanical studies have shown an increase in load to failure with inlay biceps tenodesis when compared with onlay tenodesis.^21,25,31,32^ One such study conducted by Richards et al.,^21^ using cadaver models, found a stronger load to failure using the biotenodesis screw (233N) when compared with suture anchors (135N). Despite this increased load to failure, a study of 65 patients conducted by Park et al.^14^ found that the inlay tenodesis was associated with decreased healing when compared with the onlay tenodesis. However, the authors reported that healing had no effect on the outcomes scores but did increase the rate of Popeye deformities. Conversely, Mazzocca et al.^31^ found no difference in cyclic displacement and load to failure in 20 cadaveric shoulders when comparing onlay (suture anchor) and inlay (biotenodesis screw) techniques using a suprapectoral approach.

Studies have reported a higher incidence of anterior shoulder pain associated with the suprapectoral tenodesis approach.^31^ This increased shoulder pain has been described to be a result of the biceps tendon placement within the intertubercular groove, which is lined with synovium, and a cause of tenosynovitis. The subpectoral tenodesis approach anchors avoid the intertubercular groove and its associated synovitis. Despite these findings, a recent systematic review of 409 patients comparing suprapectoral versus subpectoral biceps tenodesis showed no notable difference in VAS for anterior shoulder pain, CS, or Popeye deformities.^33^ Thus, it remains unclear whether the results of this analysis were solely because of onlay versus inlay tenodesis or the location of fixation influenced these findings.

Several limitations have been identified in this analysis. Patients included in this analysis were treated with additional procedures such as rotator cuff repairs and débridement, labral repairs and débridement, subacromial decompressions, and distal clavicle excisions, which are known to influence postoperative outcomes. Multiple studies have shown worsened proximal biceps tendon pathology with larger rotator cuff tears.^2,33,34^ In addition, multiple factors have been established to further influence the outcomes and complications that were not well-controlled in these articles. Such factors include postoperative rehabilitation, length of follow-up, prior interventions, and the anatomic location of fixation—suprapectoral versus subpectoral fixation.

## Conclusion

This meta-analysis provides evidence that both onlay and inlay biceps tenodeses for long head of biceps tendinopathy result in improved clinical outcomes and are at low risk of Popeye deformities, with no statistically significant differences between either method. Additional studies are required to assess the clinical significance of these differences.
